# Occupation for the Recent Graduate

**Published:** 1906-09-01

**Authors:** 


### OCCUPATION FOR THE RECENT GRADUATE.

What is to become of them all who year by year pass out of the schools to swell the ranks of an already crowded profession. What are they going to do? Those who have selected their parentage and social position can afford, wait, and learn and become house physicians, house surgeons, and develop into a consulting physician and surgeon or a specialist or an expert in some direction. If he has the wherewithal he may start the slow and uphill work of general practice, general practice where, as a rule, to begin leisure is plentiful and dollars scarce. All are not so favourably situated and have to look around for bread, purpose, and occupation, and in these days the search requires energy and concentration. The following are some of the directions in which the aspirant may look. He may enter the public service, clearly remembering that in doing so he foregoes practice on his own account. It must be the life work.

The attractions of the Royal Army Medical Corps and of the Indian Medical Service have recently been materially increased. In addition to improvements in pay, there is also scope for the work of a man who has special ability in some one or other branch of medicine or surgery.

The Navy.

This service is only open to doctors of European birth between twenty-one and twenty-eight years of age. He must pass an entrance examination and gain at least one-third of the possible marks. Successful candidates immediately after passing the examination will receive commissions as surgeons in the Royal Navy, and will undergo a course of practical instruction in naval hygiene, the diseases of warm climates, bacteriology, surgery of gunshot wounds, etc., radiography and the photography in connection with it at Haslar Hospital. Surgeons are only required to provide themselves with a regulation case of pocket instruments, a stethoscope, and three clinical thermometers. All other instruments are provided at the public expense.

The rate of pay of surgeons is on entry £255 10s. a year; after four years' full-pay service, £310 5s. a year; after eight years' full-pay service, or on promotion to staff surgeon, £365; after twelve years' service, £438. Fleet surgeons on promotion, special, or after twenty years in the service, £492 15s. a year; after four years' full-pay service in that rank, £547 10s.; after eight years' full-pay service, £602 5s.; and after twelve years' service, £657; Deputy Inspector-General, £766 10s. a year; Inspector-General, £1,300, consolidated. Certain allowances are also given and arrangements made for retired pay.

Every medical officer will be required to undergo a post-graduate course of three months' duration at a metropolitan hospital once in every eight years (should the exigencies of the service permit), and this as far as possible during his surgeon's, staff surgeon's, and fleet surgeon's period of service. While carrying out this course the medical officer will be borne on a ship's books for full pay, and will be granted lodging and provision allowances, and travelling expenses as for service under the regulations to and from his home or port; the fees for each course (not exceeding £25) will be paid by the Admiralty on the production of vouchers at the end of the course. The medical officer will be required to produce separate certificates of efficient attendance in the following: I. The medical and surgical practice of the hospital; II. A course of operative surgery on the dead body; III. A course of bacteriology; IV. A course of ophthalmic surgery, particular attention being paid to the diagnosis of errors of refraction; V. A practical course of skiagraphy.

### The Army.

A candidate for a commission in the Royal Army Medical Corps must be 21 years and not over 28 years of age at the date of the commencement of the entrance examination. He must possess, under the Medical Acts in force in the United Kingdom at the time of his appointment, a registered qualification to practise. He must pass an entrance examination, and, having gained a place, must undergo two months' instruction in hygiene and bacteriology, and afterwards proceed to Aldershot for instruction in technical duties of the corps. The following are the rates of pay, inclusive of all allowances except field and travelling allowances: Director-General, yearly £2,000; Deputy Director-General, £1,500; Assistant Director-General, £850; Deputy Assistant Director-General, £750. Exclusive of allowances not at head-quarters: Surgeon-General, daily £3; Colonel, £2; Lieutenant-Colonel, £1 10s.; Lieutenant-Colonel specially selected for increased pay after at least eight years' service abroad, £1 15s.; Major £1 3s. 6d., after three years' services as such, £1 6s.; Captain, 15s. 6d.; after seven years' total full-pay service, 17s.; after ten years' total full-pay service, £1 1s.; Lieutenant on probation and lieutenant, 14s.; Adjutant of the Royal Army Medical Corps (Volunteer), the pay of his rank; Quartermaster, as a quartermaster of infantry.

### Indian Medical Service.

Candidates must be natural-born subjects of His Majesty between 21 and 28 years of age at the date of the examination, of sound bodily health, and in the opinion of the Secretary of State for India in Council in all respects suitable to hold commissions in the Indian Medical Service. They may be married or unmarried. They must possess, under the Medical Acts in force at the time of their appointment, a registrable qualification to practise both medicine and surgery in great Britain and Ireland.

After passing examination, the successful candidates will be required to attend one entire course of practical instruction at the Army Medical School and elsewhere, as may be decided, in:

(1) Hygiene; (2) Military and Tropical Medicine; (3) Military Surgery; (4) Pathology of diseases and injuries incidental to Military and Tropical Service.

This course will be of not less than four months' duration.

### Preventive Medicine.

Sanitary science, or State public health or preventive medicine, is yearly increasing in scope and importance, and offers a career to men to whom any other path in the profession of medicine might prove distasteful.

A medical officer of health of any county or of any single or combined district with a population at the last census of 50,000 or more, or of any metropolitan district, or the deputy of any such officer must be legally qualified for the practice of medicine, surgery, and midwifery and also be either registered in the "Medical Register" as the holder of a diploma in sanitary science, public health, or State medicine, under Section 21 of the Medical Act, 1886, or have been during three consecutive years preceding the year 1892 a medical officer of a district or combination of districts in London or elsewhere with a population according to the last-published census of not less than 20,000, or have been, before the passing of the Local Government Act, 1888, for not less than three years a medical officer or inspector of the Local Government Board.

It is essential to obtain early the diploma of public health, and then try and obtain an assistantship to an officer in charge of a large borough or district. Diplomas are granted by nearly all examining bodies.

The Conjoint Board of England recognise the following laboratories for courses of instruction:

London.—St. Bartholomew's, *Guy's, London, University College, King's College, Charing Cross, St. George's, St. Mary's, St. Thomas's, Westminster, Middlesex.
Birmingham.—The University.
Brighton.—Technical School.
Bristol.—Medical School.
Cardiff.—University College.
Leeds.—Yorkshire College.
Liverpool.—The University; School of Science and Technicology.
Manchester.—Owens College.
Newcastle-on-Tyne.—University of Durham College of Medicine.
Sheffield.—University College.
*Bacteriology only.

The Universities, generally speaking, grant Diplomas to their own graduates or graduates of other universities.

The Conjoint Board of Scotland enables diplomates to overcome this difficulty. Candidates must produce evidence of attendance (subsequent to obtaining a registrable qualification) for six months at a Public Health Laboratory, and for six months under a Medical Officer of Health of a county or of a large urban district. There are two examinations, and candidates may present themselves for both of them at one period or for either examination separately. The fee is £12 12s., or £6 6s. in respect of each examination; and candidates referred are readmitted on a fee of £3 3s. in respect of each examination. The examination is held in Edinburgh or in Glasgow, there being two periods of examinations yearly—October and May. The examination in October, 1905, will be held in Edinburgh. Applications for examination in Edinburgh to be sent to Mr. James Robertson, 54, George Square, Edinburgh; and in Glasgow to Mr. Alexander Duncan, B.A., LL.D., 242, St. Vincent Street, Glasgow, not later than fourteen days before the examination day. The University of Dublin confers degrees after examination upon M.D.'s or graduates in Medicine and Surgery of Dublin, Oxford, or Cambridge, and the Royal University only on graduates of Medicine of that University. The Conjoint Examining Board grants diplomas after examination to candidates who have complied with the regulations of the General Medical Council.

### Books for the Study of Public Health.

The following list of books may prove of value to the student pursuing this study. They are, perhaps, the most generally appreciated, and are every one important works:

Whitelegge's "Hygiene and Public Health" (London: Cassell and Co., 1899, 7s. 6d.).

Parkes and Kenwood's "Hygiene and Public Health," second edition (London: H. K. Lewis, 1902, 12s.).

Wilson's "Handbook of Hygiene," eighth edition (London: J. and A. Churchill, 1898, 12s. 6d.).

"The Theory and Practice of Hygiene," by Notter and

Horrocks and Firth, second edition (London : J. and A. Churchill, 1900, 25s.).
Hamer's "Manual of Hygiene" (London : J. and A. Churchill, 1902, 12s. 6d.).
Stevenson and Murphy's "Treatise on Hygiene and Public Health," 3 vols., for reference on special subjects (London : J. and A. Churchill, 1892-4, 80s.).
Wynter Blyth's "Manual of Public Health" (London : Henry Renshaw, 1896, 21s.).
Kenwood's "Public Health Laboratory Work," third edition (London : H. K. Lewis, 1903, 10s. 6d.).
Wanklyn's "Water, Air, Milk, and Bread Analysis," four separate manuals (London : Kegan Paul, Trench, Trübner and Co., 5s. each).
Thresh's "Examination of Waters and Water Supplies" (London : J. and A. Churchill, 1904, 14s.).
Horrocks' "Bacteriological Examination of Water" (London : J. and A. Churchill, 1901, 10s. 6d.).
Woodhead's "Bacteria and their Products" (London : W. Scott, 1892, 3s. 6d.).
Kanthack and Drysdale's "Practical Bacteriology" (London : Macmillan and Co., 1895, 4s. 6d.).
Muir and Ritchie's "Manual of Bacteriology," third edition (London : Young J. Pentland, 1902, 12s. 6d.).
Klein's "Micro-organisms and Disease" (London : Macmillan and Co., 1896, 10s. 6d.).
Crookshank's "Text-book of Bacteriology" (London : H. K. Lewis, 1896, 21s.).
Hewlett's "Manual of Bacteriology," second edition (London : J. and A. Churchill, 1902, 12s. 6d.).
Daniell's "Text-book of the Principles of Physics" (London : Macmillan and Co., 1896, 4s. 6d.).
Balfour Stewart's "Elementary Physics" (Oxford : Clarendon Press, 7s. 6d.).
Newsholme's "Vital Statistics," third edition (London : Swan Sonnenschein and Co., 1899, 7s. 6d.).
McVail's "Vaccination Vindicated" (London : Cassell and Co., 1887, 5s.).
J. W. Moore's "Meteorology : Practical and Applied" (London : Rebman and Co., 8s.).
Scott's "Meteorology," sixth edition (London : Kegan Paul, Trench, Trübner and Co., 1893, 5s.).
Stratton's "Public Health Acts" (London : Knight and Co., 7s. 6d.).
Knight's "Annotated Model By-laws," sixth edition (London : Knight and Co., 1899, 12s.).
Corfield's "Dwelling Houses : Their Sanitary Construction and Arrangements," fourth edition (London : H. K. Lewis, 1898, 3s. 6d.).

Best Books for the D.P.H.

"The Science of Hygiene," by Walter C. C. Pakes (Methuen and Co., 36 Essex Street, W.C.).
"Applied Bacteriology," by T. H. Pearman and G. Moore (Baillière, Tindall, and Cox, 1897).

The Local Government Board's Reports afford a most valuable source of information (London : Wyman and Co.), and should be also employed.

